# Resistance against anti-CD19 and anti-BCMA CAR T cells: Recent advances and coping strategies

**DOI:** 10.1016/j.tranon.2022.101459

**Published:** 2022-05-23

**Authors:** Pinar Ataca Atilla, Erden Atilla

**Affiliations:** a Ankara University Stem Cell Institute, Ankara, Turkey; b Department of Hematology, Mersin City Hospital, Mersin, Turkey

**Keywords:** Chimeric antigen receptor T cells, Resistance, Immunotherapy, CD19, BCMA

## Abstract

Chimeric antigen receptor T (CAR T) cell therapy is a new treatment paradigm that has revolutionized the treatment of CD19-positive B cell malignancies and BCMA-positive plasma cell malignancies. The response rates are highly impressive in comparison to historical cohorts, but the responses are not durable. The most recent results from pivotal trials show that current CAR T cell products fail to demonstrate optimal long-term disease control. Resistance to CAR T cells is related to CAR structure, T cell factors, tumor factors and the immunosuppressive microenvironment. Novel strategies are needed following failure with CAR T cell treatment. In this review, we discuss the resistance mechanisms to CAR T cell treatment according to disease and the emerging strategies to overcome resistance.

## Highlights

• Some patients may experience resistance to CD19 CAR T cell and BCMA CAR T cell therapies or relapse after treatment.
• Mechanisms of resistance to CAR T cell therapies may be related to CAR structure, T cell factors or tumor associated factors.
• The strategies to overcome the resistance would allow CD19 CAR T cells or BCMA CAR T cell to be applied with a broader perspective.

## Graphical abstract

**Figure:**
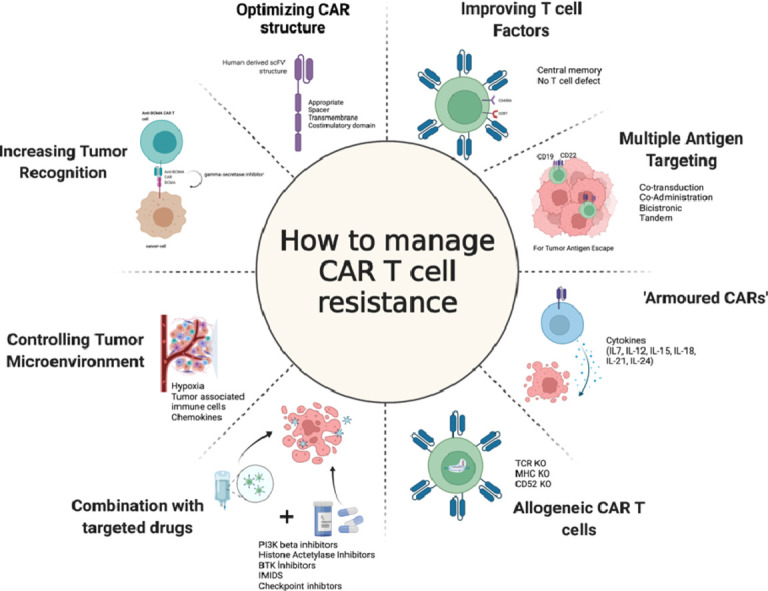

## Introduction

Chimeric antigen receptor T (CAR T) cell therapy is an immunotherapy method in which autologous T cells are reprogrammed by gene transfer to recognize tumor-associated antigens and destroy cancer cells. It was first applied by the Israeli immunologist Zelig Eshhar in 1993 and was further developed by many other researchers [1]. Producing a chimeric antigen receptor (CAR) is a complex process involving many genetic rearrangements to redirect the T cell to target cancer cells. The CAR structure contains target antigen recognition (scFv etc.), spacer (hinge), transmembrane, additional stimulator (co-stimulator) and signaling parts. T cells are activated when they bind to an antigen, independent of HLA. The affinity of the target antigen-binding part is essential for determining CAR function; too much affinity can cause cell death by activation. In addition, the density and epitope localizations of the target antigen are also important for the efficiency of CAR T cells [2,3]. The spacer part (CD8, CD28, IgG1 or IgG4) is crucial for reaching the target antigen and creating a flexible structure [4]. Transmembrane structures are responsible for stability and function generally consist of type 1 protein (CD3, CD28, CD4, CD8) [5]. Activation and persistence of T cells are due to costimulatory molecules found in second and third generation structures (e.g. CD28, CD137 (4-1BB), ICOS, CD134 (OX40), CD27 or CD244) [6].

In CD19-positive relapsed refractory B-cell malignancies, CAR T-cell therapies have become an important alternative treatment because of their significant recovery rates in patients. CD19 is a B cell-specific surface molecule that is present in all developmental stages of B cells and is also expressed on the cell surface in malignant transformation. CD19 is found in 95% of B-cell malignancies [7]. There are currently four CD19 CAR T cell therapy products commercially available. Axicabtagene-ciloleucel (KTE-019, axi-cel) was approved by the FDA in 2017 for use after two or more lines of treatment in relapsed refractory diffuse large B-cell lymphoma (primary mediastinal large B-cell lymphoma, high-grade B-cell lymphoma, and diffuse large B-cell lymphoma secondary to follicular lymphoma) [8]. Tisagenleclucel (tisa-cel) was approved in 2017 for patients with B-ALL after two or more lines of systemic therapy for ALL before the age of 25 and relapsed refractory diffuse large B-cell non-Hodgkin lymphoma (DLBCL) high-grade B-cell lymphoma, and diffuse large B-cell lymphoma secondary to follicular lymphoma in 2018 [9]. Various studies have investigated the safety and efficacy of commercially available CAR T cell therapies. A real-world experience studies on axi-cel by the Center for International Blood and Marrow Transplant Research (CIBMTR) and the US CART Consortium showed an overall response rate (ORR) between 70 and 79% and complete response (CR) rates around 50%, similar to the ZUMA-1 trial [8]. The CIBMTR registry also reported the real-world outcomes of tisa-cel in relapsed/refractory DLBCL in 70 patients with rates of ORR and CR of 59.6% and 38.3%, comparable with the JULIET trial [9]. Following the intention-to-treat ORR and CR rates of 85% and 59%, respectively with the ZUMA-2 trial, in 2020, Brexucabtagene (KTE-X19) was approved for relapsed refractory mantle cell lymphoma [10]. In 2021, Lisocabtagene maraleucel (liso-cel; JCAR017), a CD19-directed CAR T cell product incorporating a 4-1BB costimulatory domain and administered in a defined CD4:CD8 of CAR T cells, was approved for relapsed refractory non-Hodgkin lymphoma [11]. The indication of axi-cel expanded to relapsed refractory follicular lymphoma after two or more lines of systemic therapy following the ZUMA-5 trial, in which 94% of patients responded to treatment [12]. Brexucabtagene was approved recently to treat relapsed or refractory B-cell precursor acute lymphoblastic leukemia. Beyond that, that the approval of axi-cel had been widen for adult patients with large B-cell lymphoma that is refractory to first-line or that relapsed within 12 months of first-line chemoimmunotherapy due to statistically significant 4 fold greater event free survival (EFS; 8.3 months vs 2 months; hazard ratio 0.398; P<0.0001) over the current standard of care in ZUMA-7 trial [13].

In addition to the success of CD19 targeted CAR T cells in leukemia and lymphoma, many ongoing studies are targeting various antigens for broad application. B cell maturation antigen (BCMA) is an antigen found especially in malignant plasma cells, detected in very few B cells and playing a role in the survival of plasma cells. In the first phase I trial of idecabtagene vicleucel (ide-cel) (bb2121), at least partial response (PR) was achieved by 76% of the patients, including CR in 39%, with a median progression free survival (PFS) of 9 months [14]. In a phase II trial (KarMMa), 84% of the patients had triple refractory disease (refractory to one protease inhibitor, one IMiD and a CD38 antibody) and PR was achieved by 73%, including CR in 33%, and the median PFS was 8.8 months [15]. The first BCMA-targeted CAR T, ide-cel, was approved by the FDA on March 26, 2021 in patients with relapsed refractory MM who received at least four lines of treatment with immunomodulatory drugs, proteasome inhibitors and anti-CD38 monoclonal antibodies [16]. Ciltacabtagene autoleucel, a bi-epitope BCMA antigen directed CAR T cell therapy showed a promising ORR reaching 97.9% with median duration of response (DOR) of 21.8 months in CARTITUTE-1 trial receiving the FDA approval on February 28, 2022 [17]. The total number of patients treated with CAR T cell therapy is rapidly increasing with the availability of commercial products and the growing number of ongoing clinical trials.

## Mechanisms of CD19 CAR T cell resistance

A recent meta-analysis including 38 studies found that the response rate of CD19 CAR T cells was 81% in acute lymphoblastic leukemia (ALL) and 68% in lymphoma [18]. Despite these impressive results, some of patients experience primary resistance to CD19 CAR T cell treatments or relapse after infusion. Primary resistance to CAR T cells occurs in 10-20% of pediatric patients with B-ALL and around 30% in lymphoma. Relapse rates after CAR T cell therapy in B cell malignancies range from 21% to 60% as some of the major clinical trials are shown in Table 1. High tumor burden at the time of lymphodepletion has been linked with CAR T cell therapy failure in ALL and lymphoma [19,20]. Although these results are favorable in comparison to historical cohorts of heavily pretreated patients, the long-term disease-free survival was between 30% and 40% [21,22]. The prognosis is worse in patients with chronic lymphocytic lymphoma (CLL): the 18-month PFS was 29% with tisa-cel [23]. Mechanisms of resistance to CD19 CAR T cell therapies may be classified under two main categories: Target antigen positive and target negative.

**Table 1:** CD19 CAR T cell clinical trials.

**Reference**	**CAR costimulatory domain**	**Patient Population/Disease**	**Complete Remission (%)**	**Relapse post CAR T cell infusion (%)**
[25]	4-1BB	Adult/ALL	90	33
[45]	4-1BB	Pediatric and Young Adults/ALL	93	45
[135]	CD28	Adults/ALL	83	57
[46]	4-1BB	Pediatric and Young Adults/ALL	81	36
[136]	4-1BB	Adult/B Cell NHL	78	28
[137]	CD28	Adult/Diffuse large B cell lymphoma, primary mediastinal B cell lymphoma, transformed follicular lmyphoma	54	40
[23]	4-1BB	Adult/Diffuse large B cell lymphoma,	52	54
[133]	CD28	Adult/B-ALL, DLBL, follicular lymphoma, nodular HL	54	38
[62]	41BB	Pediatric/ALL	77	43
[134]	CD28-41BB	Adult/ALL and Lymphoma	50-ALL 36-Lymphoma	47

### Target antigen positive resistance

CD19 positive relapses generally occur early after initial disease remissions following CAR T cell infusion. There are three major consequences related with this: CAR structure, T cell factors and tumor factors.

#### Mechanisms of resistance associated with CAR structure

For effective CAR T cell treatment, CAR T cells must synapse to tumor cells, effectively kill the tumor cells, expand in patients and persist to eliminate tumor cells and prevent relapse. Antibodies to murine CAR scFV used in clinical studies may play a role in CAR T cell resistance. In the study of Turtle et al., murine scFV FMC63 was detected as an immunogenic epitope in five patients resistant to CAR T cell therapy [24]. Antigen-independent tonic signaling of CAR structures can limit the power of CAR T cells [25]. Selecting the appropriate spacer domain of the optimal length is critical for efficient ligand binding. The different costimulatory domains demonstrate various kinetics of anti-tumor activity. CD28-containing CAR T cells are easily activated by low antigen levels and mediate rapid initial tumor cell killing [26]. Faster activation kinetics of CD28 containing CAR T cells were associated with increased phosphorylation of CAR CD3z, Lck, ZAP-70 and LAT following *in vitro* activation [27]. This early activation leads to exhaustion and poor persistence. In the study of Zhao et al., the persistence of CD19 CAR T cells containing the 4-1BB costimulatory region was found to be longer than those containing a CD28 costimulatory region because the 4-1BB signaling domain reduced exhaustion [28].

For optimal clinical response, *in vivo* CAR T cell expansion and persistence are essential. Expansion of CAR T cells was reported to correlate with IL-6-STAT3 signaling; inhibiting these pathways decreases proliferation [29]. Additionally, the site of insertion for the CAR vector affects the CAR T cell expansion [30]. Patients with less differentiated (naïve or early memory) T cells have strong proliferative potential and resistance to exhaustion. Sustained activation of Akt in CD8 T cells promotes terminal differentiation [31]. Transcriptomic analysis showed that increased expression of genes regulating especially late memory/effector T cell differentiation and aerobic glycolysis cause poor prognosis in CAR T cell therapy [29]. At the same time, it is hypothesized that undifferentiated T cells in the T cell pool turn into more differentiated effector/memory T cells with age and decrease the efficacy of CAR T cell therapy [32].

#### Mechanisms of resistance associated with T cell factors

One of the most important factors in the response to tumor immunotherapy is the state of the patient's immune functions. The most important problem underlying the lower efficacy of CAR T cells, especially in CLL patients, is T cell defects in patients [23]. These T cell defects pose a problem in the manufacturing of autologous CAR T cells, especially in phase 1 studies (NCT01044069, NCT02445248) [33]. Exhaustion has been suggested as a major reason for T cell dysfunction. Exhausted human T cells that are related with defective c-Jun functionality have a higher number of inhibitor receptors, less proliferative potential and cytotoxity [34]. Basic leucine zipper ATF-like transcription factor (BATF) and interferon regulatory factor 4 (IRF4) counteract CAR T cell exhaustion [35].

In another study, patients who responded to CAR T cell therapy had higher polyfunctionality scores, characterized by production of multiple types of cytokines and chemokines [36]. The transduction success of CAR T cells obtained from geriatric donors is low [37]. Additionally, the number of functional T cells can be decreased related to number of prior therapies. Delays in manufacturing could be challenging in highly proliferative malignancies [38].

#### Mechanisms of resistance associated with tumor factors

Tumor cell survival and apoptosis are important in antigen-positive resistance. Tumor necrosis factor (TNF)-related apoptosis-enhancing ligand (TRAIL), Fas ligand (FasL) and cytokines such as IFN- are involved in tumor cell apoptosis [39]. Although type I cytokine secretion is normal, cytotoxic effects of CAR T cells seem to decrease when TRAIL inhibitor is administered [40]. Singh et al. demonstrated that ALL cell lines lacking the pro-apoptotic molecules FADD, BID, CASP8 or TNFRSG10 were resistant to CAR T cell killing [41]. Recently, the loss of NOXA, a B-cell lymphoma 2 (BCL2) family protein in B cell malignancies was found to be the major regulator of resistance to CAR T cell therapy by impairing the apoptosis of tumor cells [42]. Furthermore, programmed death-1 ligand-1 (PD-L1) is expressed by the tumor cells or the tumor microenvironment and inhibits the CAR T cell cytotoxicity in B cell malignancies [43].

### Target antigen negative resistance

Target antigen negative resistance (CD19 loss or downregulation) has been widely studied. Various studies have shown that CD19 negative relapses are between 9 and 25% of B-ALL cases treated with CAR T cell therapy [7,44,45]. CD19 negative recurrences have also been reported in DLBHNL (33%) [21]. Unlike TCR pathway in T cells, robust CAR T cell activation is dependent to high levels of target antigen [46], [47], [48]. A heterogeneous distribution of tumor antigens is important in CAR T cell therapy resistance. Mechanisms of antigen-negative relapses include the presence of target antigen-negative tumor cells before treatment, mutations, splicing variations, epitope masking or lineage switching [49], [50], [51], [52], [53]. Sometimes, CD19-28ζ or CD19-41BBζ CAR T cells were able to bind to target antigen however failed to activate or kill tumor cells related with the lack of presentation of target antigens [54,55]. The CD19 negative relapses were higher in patients who received CD19 directed bispecific T cell engager (BITE), blinatumomab [56].

**Fig. 1:**
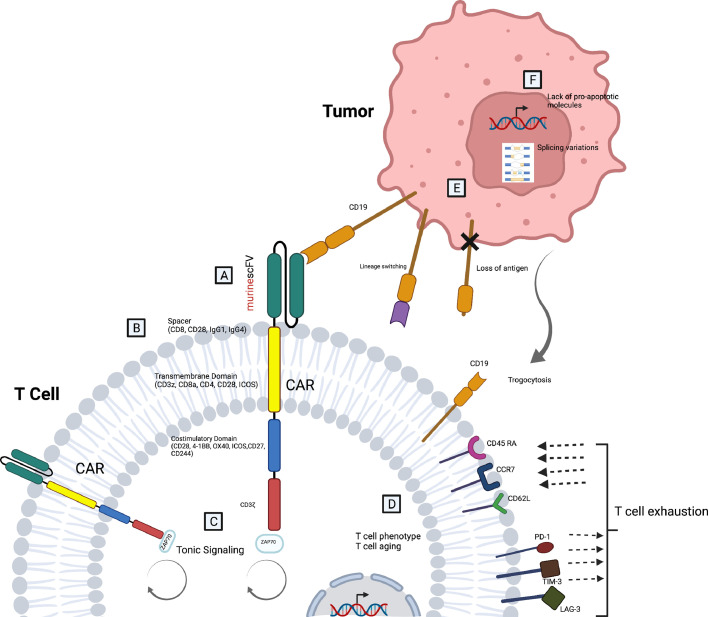
**Resistance mechanisms of CD19 CAR T cells. A.** Murine scFV is an immunogeneic epitope **B.** Different spacer and transmembrane domains effect CAR T cell efficacy. Costimulation domains regulate persistence and exhaustion **C.** Tonic signaling can limit the power of CAR T cells. **D.** T cell characteristics may effect the clinical response (T cell phenotype, T cell aging, T cell exhaustion). **E.** In antigen negative relapses loss of antigen, splicing variations, lineage swiching, trogocytosis are important mechanisms**. F.** Tumors may be lack of pro-apoptotic molecules.

In the CD19 gene, exons 1-4 encode extracellular structures, and exons 5-13 encode transmembrane structures [51]. One study examined 12 cases with CD19 negative relapse after CAR T cell therapy; it showed that especially exon 2-5 mutations - exon 2, 3 or 4 frame mutation, insertion in exon 3, or mutations in exon 4 may impair CD19 expression in the cell membrane [57]. Alternative splicing resulted the loss of the extracellular epitope of CD19 that is recognized by the CAR T cells [49]. CD19 epitope masking was reported in a relapsed B-ALL patient due to the insertion of the CAR transgene into a single leukemic B cell [58]. Gardner *et al*. showed that after infusion of CD19 CAR T cells in 7 B-ALL patients with MLL gene reorganization, recurrences occurred in two patients with myeloid phenotype after lineage switching [59]. In recent years, the concept of trogocytosis has become prominent in the mechanisms of complete and incomplete antigen escape. *In vitro* and *in vivo* experiments with CD19 positive leukemia cells demonstrated that CAR structures reversibly cause loss of antigen by trogocytosis; they transfer the target antigen to T cells. CD19 positive T cells are killed by CAR T cells (fratricide), which is effective in resistance to CD19 CAR T cell therapy [55]. The mechanisms of CD19 CAR T cell resistance are summarized in Fig. 1.

## Mechanisms to cope with CD19 CAR T cell resistance

### Optimization of CAR structure

Extensive preclinical and clinical studies have been conducted to improve the efficacy of CAR T cells due to these resistance mechanisms. There are reports that CAR T cells with human-derived scFvs have better persistence and killing power than murine scFv. A CR rate of 92.9% was achieved when CD19 CAR T cells with human scFVs were administered to refractory B ALL patients who did not receive CAR T cell therapy before. CR was obtained in only one of 3 patients who had previously received murine CAR T cells [31]. In a study by Mueller et al., although anti-murine CAR antibodies developed after tisagenlecleucel treatment were detected in 84.8% of patients, these antibodies did not change the effectiveness of the treatment [33]. Ghorashian and colleagues designed a lower affinity CD19 scFV termed ‘CAT’ to CD19 than the FMC63 sFC which improved the efficacy and prolonged the persistence [60].

CD19 CARs containing CD28 spacer and transmembrane domain were shown to secrete lower cytokine levels and reduced activation-induced cell death (AICD) compared to those containing CD8a [61]. No significant difference was observed in response rates when CD28 or 4-1BB co-stimulators were used in second-generation CAR constructs developed against CD19 in ALL [62]. In CD19 positive lymphomas, the efficacy of CAR T cells containing 4-1BB co-stimulators is slightly better than those containing CD28 co-stimulator [63]. In order to optimize CD28-CAR T activation, prevent exhaustion and create memory phenotypes, one of the ITAM motifs in CD3z can be mutated, the transcription factor c-Jun can be overexpressed, or a single residue in CD28 co-stimulation can be changed [34,64]. Additionally, the structure of CD28 can be modified for superior persistence and reduced exhaustion, as shown in a B-ALL mouse model in which both CD28 YMNM and PRRP motifs were mutated while the PYAP motif remained intact [65]. To reduce rapid CAR phosphorylation upon activation, FK 506 binding protein (FKBP) rapamycin binding (FRB) was introduced in an *in vivo* lymphoma xenograft model [18]. 4-1BB CAR T cells can be optimized to improve their initial activation kinetics by overexpression of Lck or a CD28 hinge/transmembrane domain that improves antigen recognition [27,66]. The 4-1BB containing CARs continue to signal in endosomes by mutation of intracellular lysine residues within the CAR [67]. Placing both CD28 and 41BB costimulatory domains upstream of CD3ζ in third-generation CAR T cells demonstrated variable results: some showed improved expansion, cytokine production and anti-tumor function, while others had inferior activity in comparison to second generation CARs [68,69]. The constitutively active myD88 and CD40 co-stimulation resulted in enhanced expansion and efficacy in CD19 CAR T cells [70].

### Armored CAR T cells

CAR T cell activity can be increased with the help of various cytokines (e.g.,IL-7, IL-12, IL-15, IL-18, IL-21, IL-24) or express costimulatory ligands (e.g., CD40L, 4-1BBL) that increase T cell proliferation and decrease differentiation, a strategy known as ‘armoured CARs’ or ‘TRUCKs’. IL-12 secreting CD19 targeted cord blood derived T cells retained a central memory effector phenotype, had increased antitumor efficacy *in vitro* [71] and resulted in enhanced survival in mice *in vivo* [72]. IL-15 cultured CAR T cells show increased proliferative capacity, persistence and anti-tumor activity in murine lymphoma models [73]. IL-21 promoted expansion of central memory and naïve-like T cell subpopulations, greater expression of interferon IFNγ and granzyme B, resulting in greater tumor control in murine models of B-ALL [74]. TCR deficient, human IL-18 expressing CD19 CAR T cells exhibited enhanced proliferation and antitumor activity in a xenograft model [75]. IL-24 armored CD19 CAR T cells enhanced antitumor efficacy, while improving proliferation and persistence [76]. In order to trigger the co-stimulatory molecules such as CD40, CD86 and major histocompatibility complex (MHC) class II, Kuhn *et al*. generated CAR T cells expressing CD40L [77].

### Multiple antigen targeting

With multi-antigen targeting, anti-tumor activity can be increased by providing efficacy against two or more antigens, especially in tumors with heterogeneous antigen expression. Especially, in CD19 negative or low relapses, B-cell malignancies retain other B cell markers such as CD22, CD20 or CD70a. Co-transduction, co-administration, Bicistronic CAR, SUPRA CAR, Tandem CAR, 'AND' gate CAR, and 'AND' gate CAR synNotch are some of these multiple antigen targeting strategies. In a phase 1 study conducted in patients who received CD19 CAR T cell therapy and who acquired CD19 negative recurrence or resistance, CR was obtained in 73% (11/15) of the patients with CD22 CAR T cell therapy [78]. But antigen escape was repeated; relapse with CD22- or CD22dim lymphoblasts was observed in 7 of 11 patients. Dual targeting of CD19 and CD22 in ALL was effective achieving minimal residual disease (MRD) negative in 6 of 8 patients [79].. Stanford group reported MRD negative CR in 88% of patients with ALL (*n*=17) and CR in 29% of patients with LBCL with CD19/CD20 CARs [80]. Fousek et al. showed that trivalent CAR T cells (targeted CD19, CD20 and CD22) have more effective anti-tumoral activities than CD19 CAR T cells, and at the same time, trivalent CAR T cells have been shown to form more effective immune synapses [81].

In order to overcome antigen loss or antigen mutation, universal CAR T cells derived from allogeneic healthy donors in which switch molecules between CAR T cells and tumor cells were added are on the agenda. FITC folic acid [82], chemically regulated SH2 delivered inhibitory Tail switch [83], leucine zipper domain [84], peptide neo-epitope (PNE) [85] are ‘off’ or ‘on’ safety switch systems that facilitate binding of CAR T cells to zipFvs.

### Combination of targeted therapies

Targeted drugs such as PI3K beta inhibitors, histone acetylase inhibitors, Bruton's tyrosine kinase (BTK) inhibitors, checkpoint inhibitors (PD-1 and CTLA-4), immunomodulatory drugs (IMIDs), and BCL-2 inhibitors can be administered together with CAR T cells to overcome escape mechanisms and improve anti-tumor activity [86]. Pre-clinical models suggested that lenalidomide improves the effect of CAR T cells with CD28 co-stimulatory domain by inducing phosphorylation of CD28, increasing the expression of nuclear factor kappa-light-chain-enhancer of activated B cells (NF-κB) and abrogating the inhibitory effect of cytotoxic T-lymphocyte-associated (CTLA)-4 Ig [87]. In *in vivo* Burkitt lymphoma preclinical models, CD19 CAR T cells in combination with lenalidomide had significantly decreased tumor burden and increased tumor infiltration by CD8+ T cells [88]. ZUMA-14 is a phase II clinical trial that combines axi-cel and lenalidomide in patients with refractory large B cell lymphoma [89]. Administration of ibrutinib from two weeks before leukapheresis until 3 months after CD19 CAR T cell (JCAR014) improved responses, with 88% ORR and decreased incidence of severe cytokine release syndrome (CRS) in patients with relapsed refractory CLL [55]. In parallel, 89% MRD negativity was shown when CD19 CAR T cells were administered concurrently with ibrutinib to patients who were not in CR despite at least 6 months of ibrutinib [90]. Checkpoint proteins (e.g., PD-1, PD-L1) have been shown to be upregulated after CAR T cell infusion [91]. Co-expression of CAR T cells with PD-1-blocking scFV and CAR structures or combining PD-1-blocking antibodies with CAR T cells are currently being investigated in ongoing studies [25](NCT02926833). In a phase 1/2 primary analysis of ZUMA-6, investigating axi-cel in combination with atezolizumab, a 75% ORR and 46% CR were demonstrated in relapsed/refractory DLBCL [92].

### Allogeneic CAR T cells

CAR T cells produced from allogeneic donors (‘off-the-shelf’ CAR T) seems a promising solution for use in T cell intrinsic disorders or production failures. No cases of acute graft-versus host disease (GVHD) were reported in an NCI follow-up study including 20 patients who relapsed after allogeneic stem cell transplantation and received anti-CD19 CAR T cells derived from donors. Eight of 20 achieved a response, with a 6-month PFS of 32% [93]. Various αβTCR and/or MHC class I complex expression can be altered by gene editing methods such as CRISPR-Cas9 and transcription activator-like effector nuclease (TALEN) to prevent T cell reactivity originating from allogeneic T cells from healthy, unrelated donors and to minimize GVHD [94]. Encouraging results were posted from the first in-human trial of anti-CD19 allogeneic CAR T cell therapy with TALEN-mediated TRAC and CD52 gene editing in refractory DLBC and follicular lymphoma. The ORR was 78% with 3 patients in CR, none of whom developed GVHD [95]. Recent progress in immunology has elucidated other options beside conventional αβ T cells for CAR optimization, such as NK cells, iNKT cells, γσ T cells, and induced pluripotent stem cells. CAR NK cells that do not rely on the T-cell receptor (TCR) for cytotoxic killing are another allogeneic option, and 73% of the relapsed/refractory lymphoid malignancy patients achieved clinically meaningful responses with a novel cord-blood-derived CD19-directed CAR-NK product with IL-15 support [96].

### Clinical strategies

Before CAR T cell therapy is administered, lymphodepletion regimens are applied to reduce tumor burden and regulatory T cells and increase CAR T cell response. Demethylating drugs such as cyclophosphamide (Cy), fludarabine/Cy (FC), bendamustine/pentastatin/Cy, and Flu/Ara-C (FA) are used in lymphodepletion. In a meta-analysis by Zhang *et al.*, the 6-month PFS rate was 94.6% in those who received lymphodepletion before CAR T cell therapy, while it was 54.5% (*p*<0.001) in those who did not receive lymphodepletion [97]. Flu/Cy conditioning chemotherapy augmented the levels of homeostatic cytokines and increased CAR T cell expansion and function [98].

The selection of central memory and stem cell-related memory T cells during CAR T cell manufacturing and infusion is especially important for T cell proliferation and persistence [99]. For *in vivo* CAR T cell fitness, strategies to manipulate the PI3K/Akt pathway are under investigation [100]. TET2 downregulation via an epigenetic approach was shown to promote T cells towards a central memory like state [101]. Microbiota-derived short-chain fatty acids promoted the memory CD8 T cells as well as dasatinib resulted in a memory-like phenotype [102,103].

Sequential infusion of CD19 CAR T cells is another option for relapsed patients. In the ZUMA-1 trial, patients with CD19 positive relapses were re-treated with CD19 CAR T cells. Out of 13 retreated patients, the ORR was 54% with four in CR and three in PR, and the median DOR was 81 days [6]. Gauthier and colleagues re-infused anti-CD19.BBz CAR T cells in patients with R/R leukemia and lymphoma and resulted that the re-infusion strategy was more effective among patients who received fludarabine. However, the outcomes in ALL patients were only 21% and median PFS of 4 months [104]. A larger study with longer follow-up is needed to optimize sequential CAR T cell therapy.

The effect of allogeneic hematopoietic stem cell transplantation (allo-HSCT) in patients who achieve complete remission after CAR T cell therapy is controversial. Although Summers *et al*. showed that the use of stem cell transplantation for reinforcement increased progression-free survival (*p*=0.059) [105], Watanabe et al. found no difference in progression-free survival or overall survival [3]. Zhao et al. observed similar leukemia-free survival (70.2% vs 64.1%) and OS (70.2% vs 65.4%) after a median follow-up of 4 years in patients who received allo-HSCT after achieving complete remission from CAR T therapy or after achieving CR following chemotherapy [106].

## Mechanisms of BCMA CAR T cell resistance and how to cope with them

In clinical studies of relapsed refractory MM in which BCMA-targeted CAR T cells were applied, the ORR were between 70% and 100%, while the CR was between 25% and 70%. PFS in some studies was less than 12 months, indicating myeloma recurrence [107,108]. Unlike CD19 CAR T cell treatment, investigations on resistance are still few, and not all of the mechanisms have been discovered.

### Humoral and/or cellular immune responses

The tumor binding site of the BCMA CAR is one of the sites that has been studied for resistance. In order to reduce the humoral and/or cellular immune responses against CAR T cells, human scFVs have also been used in anti-BCMA CAR T cells with orvacabtagene autoleucel. This approach increased the ORR to 92% and reduced the occurrence of severe CRS and neurotoxicity (3%) [109]. However, the development of orvacabtagene autololuecel may not be proceeded due to company decision. Similarly, fully human B-cell maturation antigen specific CAR T cells (CT053) achieved 100% CR with 50% VGPR or better with a median follow-up of 4.5 months [110]. Simplifying the CAR antigen-binding domain to remove the light-chain domain reduced the immunogenicity. Furthermore, when the CAR structure is simplified to a fully human heavy-chain variable domain (FHVH33), 4-1BB and CD3z domains mediated similar cytokine release; reduction in tumor burden compared to an identical CAR with a conventional scFv might be related to better gene expression by transduced T cells [111]. Alternative manufacturing process for delivering CAR transgene with transposon-based piggyBac is potentially less immunogenic than virus-based vector. In PRIME phase 1 / 2 study, the incidence of adverse events were lower as well as the ORR of 57% [112].

### T cell factors

Preclinical studies showed that persistence was improved if the product is rich in memory-like phenotype [99]. BB21217 is a next-generation anti-BCMA CAR T therapy uses the same lentiviral CAR T design as idecabtagene vicleucel (bb2121), but it adds the phosphoinositide 3 kinase inhibitor bb007 during *ex vivo* culture for more persistence and to enrich the product for memory-like T cells. In a 69-patient trial, the ORR was 68% (CR of 29%, median response of 17 months) [113]. In BCMA CAR T cell resistant patients, the expression of inhibitory immune checkpoint receptors such as LAG-3, TIGIT and PD-1 direct T cells to terminally exhausted and senescent stage [114]. To improve CAR T cell activity, CAR T cells can be engineered to secrete PD-1 or PD-L1 antibodies, co-transduce a PD-1/CD28 chimeric receptor, knockdown or knockout of PD-1 may be selected [115].

For broader application and to overcome the limitations due to manufacturing related with T cell fitness, allogeneic CAR T cells are increasingly popular. Sommer *et al*. demonstrated a sustained antitumor response in mice using allogeneic BCMA CAR T cells with TALEN gene editing, which was further enhanced by incorporating a CD20 mimotope-based intra-CAR off switch [116]. Allogeneic anti-BCMA CAR T cell phase I studies are ongoing (NCT04244656, NCT04171843). Initial phase 1 data of ALLO-715, a human scFV with 41BB costimulatory domain, showed promising ORR of 60% with 40% of VGFR or better without observing any graft versus host disease [117].

### Antigen loss or downregulation

Some relapses are either antigen-negative or antigen-low. Biopsy proven BCMA loss at relapse was shown in 8% of patients whereas in another cohort 67% of patients had a reduction in BCMA intensity on myeloma cells following BCMA CAR T cell infusion including 4 out of 9 non-responders [118,119]. In the KarMMa study, a loss of antigen at relapse was observed in one relapsed patient out of 16 (6%) in immunohistochemistry assessment, and serum antigen loss by soluble BCMA in approximately 4% of cases(122). A single-cell transcriptome profiling study on serially collected bone marrow samples showed a biallelic loss of BCMA in a case that represented an initial response followed by lack of response to second Idecabtagene vicleucel infusion [[120], [121]]. Gamma secretase mediated shedding from plasma cells can lead to increase of soluble BCMA. When a gamma secretase inhibitor, which increases binding to BCMA and decreases soluble BCMA, is added to anti-BCMA CAR T cell therapy, treatment efficacy is increased [109,122]. In a phase I first-in-human trial combining CAR T cells expressing a fully human BCMA scFV with an orally administered gamma secretase inhibitor, the best ORR was 100% (5 VGPR, 1PR) with 5/6 patients MRD negative [123]. Cilta-cel is another anti-BCMA CAR T designed with a bi-epitope BCMA binding that confers high-avidity binding. Preliminary results indicated that 97% of patients received at least PR with stringent CR in 67%; the response was independent of baseline BCMA expression, CAR T cell expansion and persistence [124].

There are several ways to engineer multi-specific T-cell products for antigen escape, including single bicistronic vectors expressing two CARs, tandem vectors in which a single CAR contains two binder sequences, or co-transduction of CAR T cells with two separate CAR-encoding vectors [111,125]. Several targets are studied in multiple myeloma rather than BCMA: CD19, CD38, GPRC5D, CD1 and SLAMF7. CD19/BCMA co-targeting studies showed a high overall response rate of 95% with CR rates between 16% and 57% [126]. Jiang et al. conducted a study in high-risk patients using BCMA-CD19 dual FastT CAR T cells, which showed an ORR of 93.8% with a median follow-up of 7.3 months [127]. GPRC5D is another novel target antigen expressed on all CD138-positive cells and restricted to plasma cells. When BCMA and GPRC5D were targeted together, significant survival increased in BCMA escape or two-antigen positivity compared to single-antigen targeting in *in vivo* models [128]. Dual-target CAR T expressing CD38 and BCMA achieved an ORR of 88% with a median follow up time of 9 months and PFS of 75% [129].

### Tumor microenvironement

Immunosuppressive effects arising from the tumor microenvironment are another component of resistance to treatment in MM. The tumor microenvironment consists of tumor-associated immune cells (macrophages, myeloid-derived suppressor cells, regulatory T cells (Treg)), fibroblasts, endothelial cells, extracellular cytokines, matrix proteins, and chemokines. Tumor specific activation of CAR T promotes IL-2 that upregulates T reg population. Disruption of the IL-2 axis by engineering CAR T cells to express the IL-7 receptor would reduce the Tregs and improve anti-tumor response [130]. Recent studies have been conducted to show that the expression of programmed cell death ligand 1 (PD-L1) by tumor cells triggers apoptosis in immune effector cells. Hypoxia, accumulation of lactic acid after glucose depletion, and low pH levels impair the effector function of T cells and decrease IL-2 and IFN levels. Prostaglandin E2 (PGE2) synthesized by tumor cells and antitumor activity in T cells have been shown to decrease via IL-6, chemokine ligand 1 (C-X-C motif, CXCL1) and granulocyte colony stimulating factor (G-CSF) pathways [131]. To overcome the inhibitory effects from the tumor microenvironment: ‘Armored’ CAR T cells can be engineered to secrete immune-stimulatory cytokines, immune-suppressive signals can be inhibited or genes encoding inhibitory signals can be removed [115]. In MM, urokinase-type plasminogen activator receptor (uPAR) was reported to increase cancer-associated fibroblasts during disease progression, and anti-uPAR CAR T was shown to ablate the cells *in vitro* and *in vivo* and restore tissue homeostasis in mice with liver fibrosis [132]. The resistance mechanisms to BCMA CAR T cells and the strategies to overcome them are summarized in Table 2.

**Table 2:** The resistance mechanisms to BCMA CAR T cells and the strategies to overcome them.

**Resistance Mechanisms to BCMA CAR T cells**	**How to improve CAR T Therapy?**
Humoral and/or cellular immune responses	Fully human scFVs or heavy-chain variable domain
Antigen loss or downregulatation	Increase antigen density (e.g. gamma-secretase inhibition) or bi-epitope binding
Impaired CAR-T expansion/persistance	Multi-antigen targeting (Dual target, OR-target)
Immunosuppression by bone marrow microenvironment	Combination of immunomodulatory agents

## Conclusion

Anti-CD19 and anti-BCMA CAR T cell therapy is a breakthrough advance in malignant hematology and has dramatically changed the treatment landscape. Nonetheless, long term benefit can be achieved in half of the patients [[18], [107]]. Despite intensive efforts, CAR T cell resistance remains an important drawback. Various studies have evaluated the resistance mechanisms associated with CAR-T cell infusion: lack of CAR T cell persistence, T cell exhaustion, target antigen escape, lineage switch, genetic mutations, factors related with tumor and tumor microenvironment [[49], [50], [51], [52], [53], [108], [130], [135]]. Strategies to overcome the resistance or relapse following CAR T cell infusions are optimizing CAR design, sustaining T cell fitness, endorsing optimal manufacture conditions, targeting multiple antigens, switching from autologous products to universal, safe and potent allogeneic products, combining pharmaceuticals to fight with microenvironmental negative effects [[78], [86], [93], [130]]. Eventually, evidence of enhanced potential of CAR T cells will shape the future and allow broader applications in B cell malignancies.

## Funding

None.

## CRediT authorship contribution statement

**Pinar Ataca Atilla:** Conceptualization, Data curation, Methodology, Resources, Software, Writing – original draft, Writing – review & editing. **Erden Atilla:** Conceptualization, Data curation, Methodology, Resources, Software, Writing – original draft, Writing – review & editing.

## Declaration of Competing Interest

The authors declare that they have no known competing financial interests or personal relationships that could have appeared to influence the work reported in this paper.
